# Isolation of high‐quality RNA from intervertebral disc tissue via pronase predigestion and tissue pulverization

**DOI:** 10.1002/jsp2.1017

**Published:** 2018-06-19

**Authors:** Stephanie Caprez, Ursula Menzel, Zhen Li, Sibylle Grad, Mauro Alini, Marianna Peroglio

**Affiliations:** ^1^ AO Research Institute Davos Davos Switzerland

**Keywords:** gene expression, intervertebral disc, real‐time PCR, RNA isolation

## Abstract

The isolation of high‐quality RNA from the intervertebral disc and especially from the nucleus pulposus is challenging due to the low cellularity and high proteoglycan content of this tissue. In this study, we report a simple modification of the standard guanidinium thiocyanate‐phenol‐chloroform extraction method, which involves enzymatic predigestion of the tissue prior to standard RNA isolation. Yield, purity and integrity of RNA isolated from bovine nucleus pulposus, inner annulus fibrosus and outer annulus fibrosus were compared among complete matrix digestion, predigestion and pulverization, pulverization alone, and pulverization followed by on‐column purification. With predigestion, the average yield of RNA obtained from bovine nucleus pulposus was 8.82 ± 2.05 ng/mg of wet tissue with 260/280 and 260/230 optical density ratios of 1.91 ± 0.15 and 1.84 ± 0.30, respectively. RIN analysis indicated that RNA quality was best preserved with the predigestion method (RNA integrity number > 7), and the extracted RNA was suitable for real‐time polymerase chain reaction. This method is of importance for gene expression studies on intervertebral disc development, degeneration and repair, and we anticipate that it may be further applied to other tissues rich in proteoglycans.

## 1 INTRODUCTION

The intervertebral disc (IVD) is composed of an outer fibrotic tissue rich in type I collagen (annulus fibrosus, AF) and a central jelly core rich in proteoglycans and type II collagen (nucleus pulposus, NP). The AF itself can be separated in outer and inner AF, with the latter being a transition zone between the outer AF and the central NP. While there is a natural evolution of the IVD macroscopic structure and phenotype during life, IVD degeneration is considered to be an accelerated aging process of the IVD and has often been associated to low back pain.^1^ Since the repair capacity of the IVD is limited, tissue engineering strategies for IVD repair have been extensively investigated and hydrogels have been suggested for NP repair, while fibrous structures have proven to be beneficial for AF restoration.^2^ Most recent strategies aim at rejuvenating the aging disc by the use of stem cells^3^, ^4^ and at enhancing the endogenous repair of the disc by injecting an appropriate stimulating factor.^5^ Gene expression analysis plays an important role in the assessment of the success of IVD repair strategies as it can provide useful information about cellular mechanisms of tissue repair and restoration of a healthy cell phenotype. Besides, such analyses are also used to investigate the phenotype of the IVD cells, including transcriptome studies. Gene expression analyses rely on the isolation of high‐purity RNA from disc tissue. However, the isolation of RNA from the IVD, and especially from the central jelly‐like NP tissue, is particularly challenging due to a unique combination of low cellularity (~4000 cells/mm^3^) and high proteoglycan content.^1^ One notable proteoglycan, aggrecan, tends to coprecipitate with RNA during the RNA isolation process.^6^

Considering the high amounts of proteoglycans present in cartilage, several protocols have been specifically developed for the successful isolation of RNA from cartilage.^7^, ^8^, ^9^, ^10^ There have been other techniques described for the isolation of RNA from disc tissue which can avoid the problem of proteoglycan coprecipitation, such as enzymatic tissue digestion,^11^ although the yields from single discs are relatively low. Additionally, specific protocols have been developed for the isolation of RNA from cells cultured in polysaccharide‐based hydrogels.^12^, ^13^ However, these techniques may not be as successful when applied to disc tissue, which has an even higher proteoglycan content and lower cellularity. Recently, Lee et al have proposed cryosectioning, second phase separation and high salt precipitation to obtain RNA suitable for real‐time polymerase chain reaction (RT‐PCR) from IVD tissues.^14^ In another study, Peeters et al have suggested to disrupt and homogenize the tissue using rapid agitation of ceramic balls in the presence of the lysis buffer, followed by proteinase K digestion and purification with a silica column.^15^ However, the 260/230 ratio obtained by these methods is relatively low, suggesting the presence of contaminants (proteoglycans, extraction buffer) or degraded RNA.

Our goal was to develop a method for isolating high‐quality RNA from all disc tissues that allows using small amounts of tissues (approximately 100 mg per sample). Indeed, the amount of tissue available can be limited when caudal discs from large animals or IVDs from small animals (eg, mouse, rat, rabbit) are used. In addition, the number of samples to process from a single experiment can be relatively high when NP, inner AF and outer AF are collected from multiple discs. In this study, we compare 3 previously established methods (complete tissue digestion, pulverization only, pulverization and on‐column purification) for the isolation of RNA from bovine disc tissue to a new protocol (predigestion and pulverization) that produces reliable amounts of high‐quality RNA. This new method is based on predigestion of the tissue with pronase prior to extraction with guanidinium thiocyanate‐phenol‐chloroform. We demonstrate that this method is valid for the isolation of RNA from all tissues of the IVD, namely NP, inner and outer AF.

## 2 MATERIALS AND METHODS

### 2.1 Intervertebral disc samples

IVDs comprising cartilaginous endplates were harvested from bovine tails (10‐12 months old) obtained from a local abattoir within 2 hours of death and washed in phosphate buffered saline (PBS) containing 10% penicillin‐streptomycin (Gibco, Zug, Switzerland) for 10 minutes, followed by a second wash in PBS with 1% penicillin‐streptomycin. Three bovine tails were dissected, and the 2 biggest discs were collected from each tail. IVD health state was confirmed by visual inspection by an experienced scientist. Thickness of AF (normal or not), jelly behavior of NP (quick shape recovery following deformation induced with a spatula) and absence of vasculature ingrowth inside the IVD were assessed. Discs were cultured overnight in Dulbecco's modified Eagle's medium (DMEM 4.5 g/L glucose) with 2% fetal bovine serum (FBS, Gibco), 1% insulin transferrin selenium and 0.2% Primocin (Invivogen, Nunnigen, Switzerland). From each disc, NP, inner and outer AF tissues were collected and a piece (100‐150 mg) of each tissue type was randomly assigned to 1 of the 4 RNA isolation groups. Tissue from each disc and tail was treated separately (*n* = 6). For RNA isolation, NP and inner and outer AF tissues from each disc were separated and chopped into small pieces (1−3 mm^3^) using a scalpel. The minced tissues were stored in 5 mL PBS/sample to avoid tissue drying until all samples were prepared. The tubes containing the minced tissues were briefly centrifuged (500*g*, 2 minutes) and PBS was aspirated.

### 2.2 Complete matrix digestion method

Minced tissue samples (*n* = 6 per tissue type) were digested using a two‐step enzymatic digestion (pronase followed by collagenase), as previously described.^11^ Briefly, tissue was digested at 37°C on a waving shaker (Polymax 1040, Heidolph, Schwabach, Germany) set at 40 rpm for 1 hour in DMEM containing 2 mg/mL pronase (Roche, Mannheim, Germany). The volume of pronase solution was adjusted to the amount of tissue collected (5 mL of pronase solution for 100 mg of tissue). The tissue was then centrifuged at 300*g* for 2 minutes and washed twice with PBS, followed by a second digestion (3 hours for NP, 4 hours for inner and outer AF) at 37°C on a waving shaker set a 40 rpm using collagenase II (Worthington, Ohio) at a concentration of 150 U/mL in DMEM. The volume of collagenase II solution was 5 mL per 100 mg of original tissue wet weight. Digestion was terminated by adding FBS (0.5 mL FBS/5 mL solution) when the tissue appeared macroscopically nearly digested. The cell suspension was filtered through a 70 μm cell strainer, cells were washed twice (with 7 minutes centrifugation step at 300*g* in between) with DMEM and lysed in 1 mL of TRI Reagent and 5 μL of polyacryl carrier (both products from Molecular Research Center, Cincinnati, OH, USA).^16^ RNA isolation continued according to the protocol of the manufacturer, where 1‐bromo‐chloro‐propane (0.1 mL per 1 mL TRI Reagent; Sigma, Buchs, Switzerland) was added resulting in an aqueous layer, from which RNA was obtained by precipitation with 0.25 mL isopropanol and 0.25 mL high salt precipitation solution (Molecular Research Center). The RNA pellet was washed with 75% ethanol, followed by 70% ethanol, prior to dissolution in 30 μL diethylpyrocarbonate‐treated water.

### 2.3 Predigestion and pulverization method

**Figure 1:**
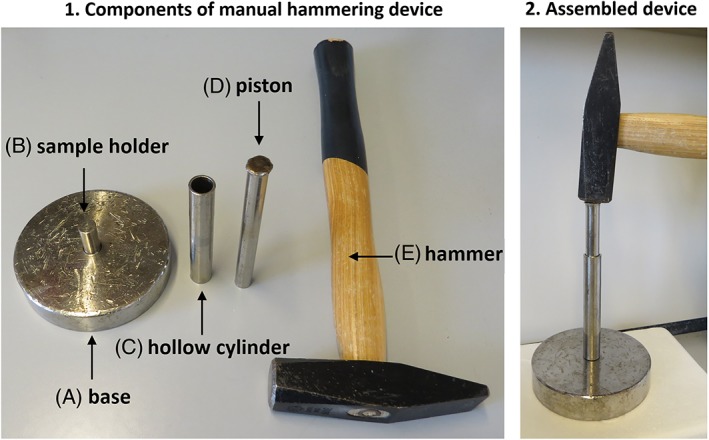
(1) Components of the manual hammering device and (2) assembled device. The sample holder is screwed on the base. Prior use, components (A) to (D) are cooled in liquid nitrogen; the piston is kept in liquid nitrogen until use. Component (C) is mounted on component (A + B) and liquid nitrogen is poured inside the hollow cylinder. The frozen sample is transferred into the hollow cylinder and more liquid nitrogen is poured. The cooled piston is added on top of the sample and the frozen tissue is hammered 5 times (a tablet is obtained). The hollow cylinder with the sample is turned upside down, more liquid nitrogen is added, and the sample is hammered 5 times again. The sample tablet is then pushed onto the base a by gentle hammering. The tablet of powdered tissue is transferred into a 2 mL Eppendorf tube with 1 mL of TRI Reagent and 5 μL polyacryl carrier. The tubes are opened and closed quickly a few times until no nitrogen gas is left and then vortexed for 5 seconds

Minced tissue samples (*n* = 6 per tissue type) were digested at 37°C for 1 hour in DMEM with pronase using the same amounts and concentrations as specified above. The digestion was then stopped with FBS and the tissue was washed twice with PBS. Any remaining PBS was aspirated, and tissue was snap‐frozen and pulverized in liquid nitrogen with a custom‐made pestle device (Figure 1). Pulverization was performed in the frozen state in the presence of liquid nitrogen and lasted few seconds. Powdered samples were transferred in the frozen state to 2 mL microcentrifuge tubes containing 1 mL TRI Reagent and 5 μL of polyacryl carrier and homogenized using a Tissue Lyzer (Qiagen, Switzerland) at 25 Hz for 6 minutes with a single 8 mm diameter stainless steel ball. Following centrifugation at 2000*g* for 2 minutes to remove remaining tissue debris, the supernatant was transferred to a 1.5 mL microcentrifuge tube and RNA isolation continued as described above.

### 2.4 Pulverization only method

Minced tissue samples (*n* = 6 per tissue type) were frozen and pulverized in liquid nitrogen with a custom‐made pestle device. The resulting powder was mixed with 1 mL TRI Reagent and 5 μL of polyacryl carrier and homogenized using a Tissue Lyzer as described above. Following centrifugation at 2000*g* for 2 minutes to remove remaining tissue debris, the supernatant was transferred to a 1.5 mL microcentrifuge tube and RNA isolation continued as described above.

### 2.5 Pulverization and on‐column purification method

Minced tissue samples (*n* = 6 per tissue type) were frozen and pulverized in liquid nitrogen with a custom‐made pestle device. The resulting powder was mixed with 1 mL TRI Reagent and 5 μL of polyacryl carrier and homogenized using a Tissue Lyzer. Following centrifugation for at 2000*g* 2 minutes to remove remaining tissue, the supernatant was transferred to a new 1.5 mL tube. RNA isolation continued as described above until phase separation: the resulting aqueous layer was mixed with pure ethanol (0.7 mL ethanol per 1 mL of acqueous phase), transferred to an RNeasy column (Qiagen) and cleaned according to manufacturer's instructions. RNA was eluted with 30 μL of RNase‐free water.

### 2.6 RNA concentration and purity analysis

RNA obtained from the 4 different methods was analyzed spectrophotometrically from 230 to 400 nm (NanoDrop 1000, ThermoFisher Scientific, Reinach, Switzerland). Since nucleic acids absorb at 260 nm, it is possible to calculate the concentration of RNA in a solution based on Beer‐Lambert's law. With a 1‐mm path length, an absorbance of 1.0 at 260 nm corresponds to 40 μg/mL of RNA. Contamination of RNA by other molecules (eg, proteins, organic compounds) is assessed by measuring the absorbances at 230 and 280 nm. The ratios of the absorbances at 260 and 280 nm (260/280 ratio) and the ratios of the absorbances at 260 and 230 nm (260/230 ratio) are used as indicators of RNA purity.

### 2.7 RNA integrity number analysis

To complete the results obtained spectrophotometrically, a RNA integrity number (RIN) evaluation was conducted on a separate set of samples (*n* = 2 per group). The RNA concentration and purity (260/280 ratio) of the samples were determined spectrophotometrically and samples were sent to Microsynth AG (Balgach, Switzerland) for RIN analysis. Samples were quantitatively assessed using an Agilent 2100 Bioanalyzer System and Agilent 2100 expert software (Agilent Technologies, Santa Clara, CA, USA).^17^ The Bioanalyzer software provided the ratio of the 18S to 28S ribosomal subunits by applying an algorithm to the whole electropherogram. The RIN was used as an indicator of RNA quality (scale from 1 to 10, with 1 strongly degraded RNA and 10 intact RNA).

### 2.8 Gene expression analysis

Samples with too low RNA quantity or quality were excluded from gene expression analysis. Criteria for inclusion were defined as follows: RNA concentration >7 ng/μL and 260/280 absorption ratio >1.6 (under these conditions, the 260/230 absorption ratio does not have a strong effect on the RT‐PCR outcome). For all samples meeting these requirements, reverse transcription was performed using TaqMan reverse transcription reagents (TaqMan RT buffer, 5.5 μM MgCl_2_, 500 μM each deoxynucleotide triphosphate (dNTP), 2.5 μM random hexamers, 0.4 U/μL RNase inhibitor, and 1.35 U/μL Multiscribe reverse transcriptase; Applied Biosystems, Forster City, CA, USA). For all samples, 500 ng of total RNA was transcribed in 20 μL reaction mixture and the resulting cDNA was diluted 1:4 Tris‐ethylenediaminetetraacetic acid (Tris‐EDTA) buffered RNase‐free water. For all samples, real‐time PCR was performed using TaqMan Gene Expression Master Mix (Applied Biosystems) with QuantStudio 6 Flex instrument (Applied Biosystems). The following genes were analyzed: collagen type Iα1 (*COL1*), collagen type IIα1 (*COL2*), aggrecan (*ACAN*), matrix metalloproteinase 3 (*MMP3*) and a disintegrin and metalloproteinase with thrombospondin motifs 4 (*ADAMTS4*). The 18S ribosomal RNA (*18S*) and glyceraldehyde 3‐phosphate dehydrogenase (*GAPDH*) were used as endogenous controls. Primers and TaqMan probes were supplied by Microsynth or Applied Biosystems, using the same sequences as in previous studies.^18^, ^19^

### 2.9 Statistical analysis

Since the tissues (NP, inner AF, outer AF) collected from 1 disc were split into 4 groups (the 4 RNA isolation methods) and 6 discs were used in the study, a two‐way analysis of variance (ANOVA) with subject matching and Tukey post‐hoc was performed (*P* < .05 was considered significant) for RNA yield, concentration, 260/280 and 260/230 ratios. A one‐way ANOVA with subject matching and Tukey post‐doc (*P* < .05 was considered significant) was performed on the Bioanalyzer data and corresponding Nanodrop data (data from different tissues using the same RNA isolation method were combined in one group). For gene expression analyses, since not all genes of interest were detected in all samples, a two‐way ANOVA without subject matching was performed with Tukey post‐hoc (*P* < .05 was considered significant).

## 3 RESULTS

**Figure 2:**
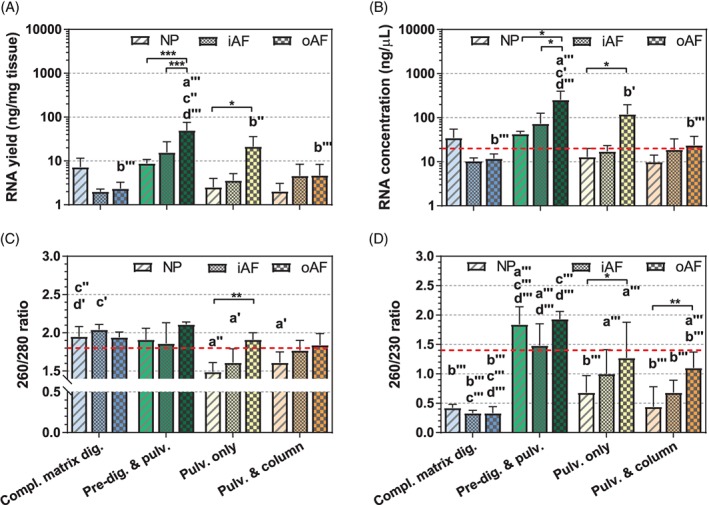
(A) RNA yield, (B) concentration, (C) 260/280 ratio, and (D) 260/230 ratio obtained for nucleus pulposus (NP, stripes), inner AF (iAF, small squares), and outer AF (oAF, big squares) tissues of bovine intervertebral discs by using different extraction methods: complete matrix digestion (“Compl. matrix dig.”, in blue), predigestion and pulverization (“Pre‐dig. & pulv.”, in green), pulverization only (“Pulv. only”, in yellow) and pulverization and on‐column (“Pulv. & column”, in orange). Data are represented as mean ± SD; *n* = 3 to 6. Red dotted line represents the suggested acceptable threshold minimum value for each parameter. Statistical analysis of the RNA extraction method for each tissue type: “a” vs complete matrix digestion, “b” vs predigestion and pulverization, “c” vs pulverization only, and “d” vs pulverization and on‐column; ′<.05, ″<.01, ‴<.001. Statistically significant differences among tissues processed with the same RNA extraction method are represented with a line; * <.05, ** <.01, *** <.001

**Figure 3:**
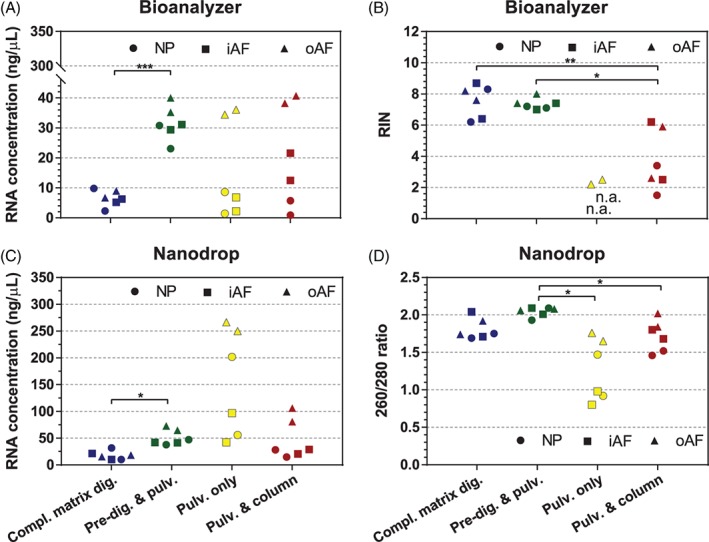
RNA concentration and RNA integrity number measured with Bioanalyzer, and RNA concentration and 260/280 ratio measured by Nanodrop for NP, inner AF (iAF) and outer AF (oAF) of bovine discs, according to the method used to isolate the RNA from each sample. Results of both duplicates are provided (n.a., not applicable); * <.05, ** <.01, *** <.001

The yield, concentration and purity of the RNA obtained with the 4 RNA isolation techniques are summarized in Figure 2 (and Table S1, Supporting Information). Since pure RNA absorbs at 260 nm, the 260/280 and 260/230 absorption ratios are often used as indicators of RNA purity. For NP tissue, highest RNA concentrations were obtained with predigestion and pulverization, followed by complete matrix digestion, while pulverization (alone or in combination with on‐column purification) gave the lowest RNA concentrations (<15 ng/μL). The highest 260/280 absorption ratio was observed with complete matrix digestion and predigestion and pulverization (1.95 ± 0.13 and 1.91 ± 0.15, respectively), pulverization with on‐column purification (1.61 ± 0.14) and simple pulverization (1.49 ± 0.12). A 260/230 absorption ratio above 1.5 was only obtained with predigestion and pulverization, while it was in the range 0.4 to 0.7 with all other techniques. For inner AF tissue, highest RNA concentrations were found with predigestion and pulverization, while all other techniques led to relatively low RNA concentrations. The 260/280 and 260/230 absorption ratios obtained for the inner AF followed a similar trend to the ones of NP tissue. For outer AF tissue, highest RNA concentrations were obtained by predigestion and pulverization, followed by pulverization alone, while complete matrix digestion and pulverization with on‐column purification led to low RNA concentrations. For this tissue, the 260/280 absorption ratio was above 1.8 with all 4 RNA isolation techniques, but the 260/230 absorption ratio was above 1.5 only with the predigestion method. The average RIN was >7 for all tissues prepared with either complete matrix digestion or predigestion and pulverization. Samples prepared with the latter technique showed the least variation in RIN (consistently >7 for all tissues and replicates), while there was a greater variability in RIN for the samples prepared by complete matrix digestion (Figure 3 and Table S2). Pulverization resulted in strong RNA degradation, which could only partially be recovered by further purification with a column (RIN < 3 for NP and RIN < 5 for AF). Interestingly, the predigestion and pulverization resulted in the lowest variability in RNA concentrations for all tissue types, highest RNA concentration (measured by Bioanalyzer) with lowest variability among replicas, and highest 260/280 ratios (Figure 3 and Table S2). The differences in RNA concentration values obtained by the Nanodrop and Bioanalyzer instruments can likely be attributed to the different measurement methods. While Nanodrop is based on UV absorbance measurement of the entire sample, the Bioanalyzer first separates biological molecules (DNA, RNA, proteins) and uses a laser method to detect/quantify the RNA.

**Figure 4:**
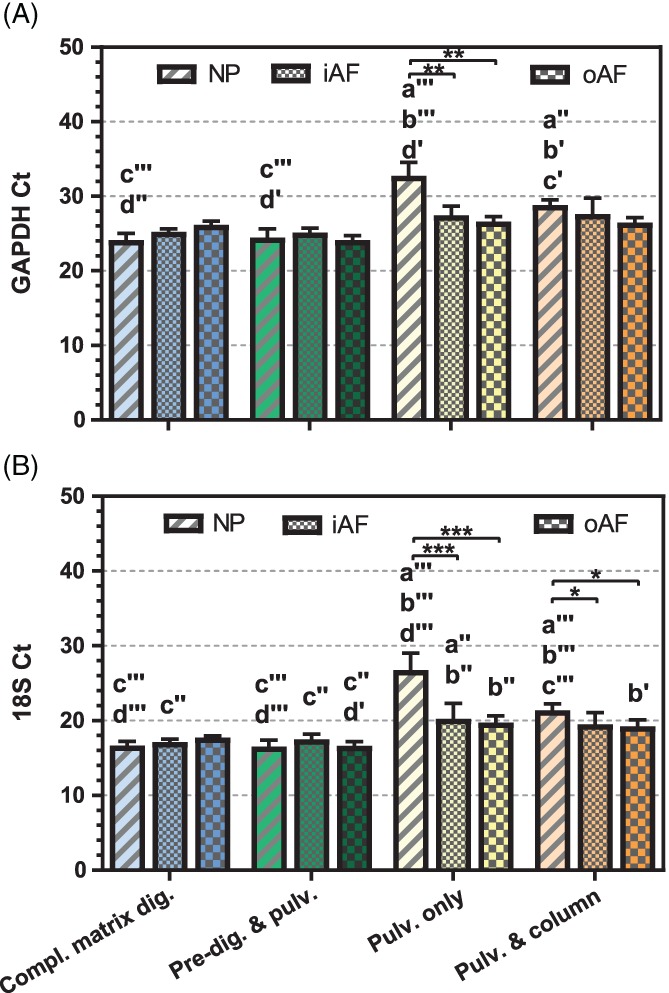
Real‐time polymerase chain reaction Ct values of endogenous control genes for the NP, inner AF, and outer AF tissues of bovine discs, according to the method used to isolate the RNA from each sample. Data are represented as mean ± SD; *n* = 3 to 6. Statistical differences are represented as described in Figure 2

To further validate the quality of the RNA obtained from these 4 methods, RT‐PCR was performed. First, 2 endogenous controls were analyzed (Figure 4 and Table S3). *GAPDH* and *18S* were detected at later cycles of amplification for samples obtained by pulverization alone and pulverization and on‐column purification compared to the samples obtained by complete matrix digestion as well as predigestion and pulverization, especially in the case of NP tissue.

**Figure 5:**
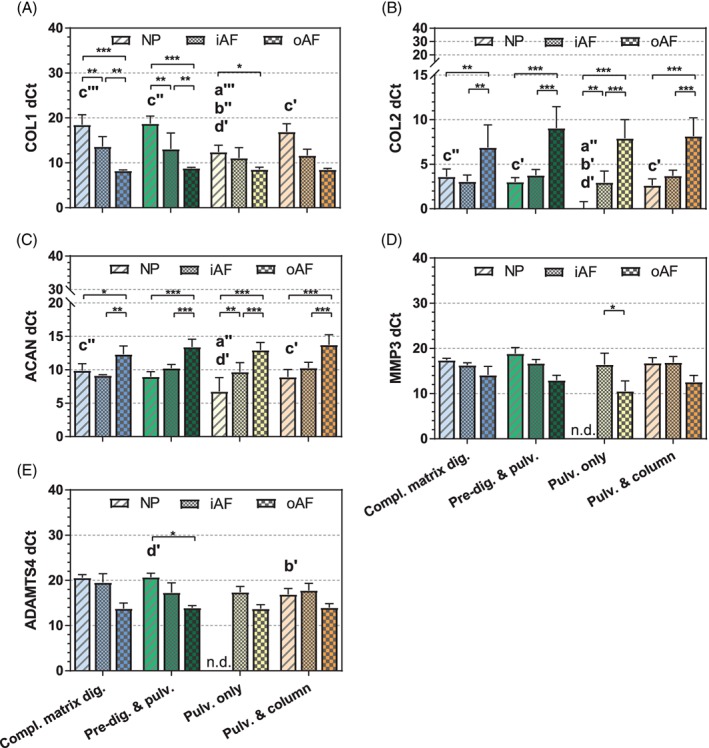
Real‐time polymerase chain reaction dCt values of genes of interest for the NP, inner AF, and outer AF tissues of bovine discs, according to the method used to isolate the RNA from each sample. *18S* was used as endogenous control; data are represented as mean ± SD; *n* = 3 to 6; n.d., gene not detected. Statistical differences are represented as described in Figure 2

Threshold cycle (Ct) values of the anabolic genes collagen type I, collagen type II, and aggrecan, and catabolic genes *MMP13* and *ADAMTS4*, which are normally expressed in disc tissue, were measured and normalized to the Ct values of the housekeeping genes *GAPDH* and *18S* rRNA. While all 5 genes could be detected, the dCt (delta‐Ct) value for each gene differed depending on the RNA isolation method used, especially in the case of NP tissue (Figure 5 and Table S4). Using the dCt method, a low dCt value indicates a high expression of the respective target mRNA. Therefore, our results are completely in line with the expected trends. For all the extraction methods, lowest *COL1* expression levels were found in the NP, while highest levels were observed in the outer AF. In contrast, *COL2* and *ACAN* showed lowest expression values in the outer AF. This indicates that large gene expression differences of around one order of magnitude (dCt > 3) are likely to be identified by all the extraction methods compared in this study.

## 4 DISCUSSION

The IVD is composed of 2 tissues, the central NP rich in proteoglycans and the AF rich in collagen. Ideally, the chosen RNA isolation method should allow the isolation of high‐quality, intact RNA from all tissue types. Several methods have been reported for the isolation of RNA from collagen‐rich tissues such as cartilage,^7^, ^8^, ^9^, ^10^ and polysaccharide‐rich materials such as plants and chitosan‐based hydrogels.^12^, ^13^ The main challenge of isolating RNA from IVD (and NP in particular) is the combination of low cellularity and high proteoglycan content. In the following paragraph, the effects of the homogenization, lysis buffer and RNA extraction method are discussed in detail.

In terms of homogenization methods of IVD tissues in preparation for RNA isolation, Peeters et al have shown that RNA of higher purity is obtained when goat NP tissue is first pulverized in liquid nitrogen and then suspended in lysis buffer rather than homogenized in lysis buffer directly.^15^ Similarly, Lee et al recommend cutting the tissue into cryosections prior to homogenization in lysis buffer. From our experience (data not shown), we can confirm that pulverization of NP frozen tissue prior to lysis gives higher RNA yields compared to homogenization in lysis buffer directly.^14^ Pulverization in liquid nitrogen can be performed either manually with an inexpensive, custom‐made device (Figure 1), or semiautomated with a cryostat^14^ or in an automated way by purchasing a commercially available homogenizer (eg, Freezer Mill, Spex CertiPrep, Polytron).^15^, ^20^ The device represented in Figure 1 might represent a simple option to more complex (and expensive) instruments. This device is suitable for small samples (100‐150 mg) as there is virtually no tissue loss. We hypothesize that a similar efficiency is obtained with either of these methods.

As for the lysis buffer, Peeters et al have shown that RNeasy Fibrous Tissue kit (Qiagen, Hombrechtikon, Switzerland) yields RNA of higher purity (as attested by 260/280 ratio) compared to RNA isolation with TRIzol.^15^ However, both the mean 260/230 ratio and the RIN value were low (<0.5 and <5, respectively), which may indicate the presence of impurities and/or degraded RNA. In another study, Lee et al have obtained a four times higher RNA yield using the classic TRIzol method compared to isolation with TRIzol followed by purification of the aqueous phase using a column‐based method.^14^ Of note, RNA yield was very low using the RNeasy column (cell lysis in RLT buffer provided in the Qiagen RNeasy kits followed by on‐column purification). In our hands (data not shown), phenol‐guanidine isothiocyanate‐based solutions are superior for lysis of NP tissue compared to RLT buffers (Qiagen RNeasy kits) and cetyl trimethylammonium bromide.^12^ Interestingly, the RNA extraction method suggested by Peeters et al involves an enzymatic digestion step with proteinase K after the homogenization and it was suggested in this study that a higher volume of enzymatic solution improves both the yield and quality of the extracted RNA.^15^ This supports our finding that a short enzymatic digestion is an important step when isolating RNA from IVD tissues. In our hands, this step is most beneficial when performed prior sample homogenization in the lysis buffer (data not shown).

Concerning the RNA extraction step, Lee et al have recommended to add a second phase separation and to use a high salt solution for precipitation.^14^ We have found that a second phase separation is not required when a pronase predigestion step is performed prior RNA extraction and that the use of a high salt solution for RNA precipitation does not seem to have a major influence on RNA yield or purity when pronase predigestion is performed. If the phase separation is suboptimal, we recommend mixing the sample again, split it into 2 tubes, add 0.5 mL of TRIzol to each tube and continue with the usual RNA isolation procedure for TRIzol samples. At the end of the RNA extraction, the 2 tubes from the same samples are pooled (the pellet in the first tube is re‐hydrated and the solution transferred to the second tube).

It is worth noting that only with the pronase predigestion step, the 260/230 ratio of RNA isolated from bovine NP was ~1.5, while even with extra phase separation and use of high salt solution the 260/230 ratio was <0.8 (RIN not reported).^14^ Therefore, we conclude that the pronase predigestion is a key step to obtain not only samples of high yield but also of high purity in all terms: RIN, 260/280 and 260/230 ratios. Pronase is a mixture of several nonspecific proteases that digest proteins down to single amino acids. Pronase is often used prior collagenase treatment for isolation of IVD cells. We hypothesize that pronase will cause partial extracellular matrix (ECM) loosening. Hence, some ECM will be removed already during the washes and centrifugation steps prior sample freezing. Moreover, pronase predigestion may contribute to a better exposure of the cells to the lysis buffer, which will in turn help to achieve a good phase separation and reduce the risk of coprecipitation of proteoglycans with RNA during the RNA extraction process.

In summary, we recommend:

- 100 to 150 mg tissue per sample
- predigestion in 0.2% pronase (5 mL/100 mg tissue) for 1 hour at 37°C on a shaker
- pulverization of IVD tissues in liquid nitrogen
- lysis in TRIzol (1 mL/100 mg of initial tissue weight)
- homogenization (25 Hz, 6 min)
- 1 phase separation
- precipitation with isopropanol and high salt solution
- 2 washing steps with 70% ethanol

Our results are similar to the data reported by Yu et al on RNA isolation from cells encapsulated in chitosan hydrogels, confirming that the use of an enzymatic pretreatment improves the purity of samples isolated with guanidinium thiocyanate.^13^ In contrast to this previous work, enzymatic pretreatment with pronase resulted in good yield, and high 260/280 and 260/230 absorption ratios without the need of further purification using RNA columns. This difference could be explained by the difference in the material (polysaccharide hydrogels vs NP tissue) and enzyme used (lysozyme vs pronase).

Besides RNA extraction directly from IVD tissues, complete tissue digestion leading to a single cell suspension has been widely used. In the present study, isolation of good quality RNA (RIN > 7) was possible with either complete matrix digestion or predigestion and pulverization. Between these 2 methods, more consistent RIN values were obtained with the predigestion and pulverization method, probably because the endpoint of complete digestion must be monitored and adapted (eg, discs from older animals require longer digestion times), which can result in under‐ or over‐digestion, thereby affecting both RNA yield and quality. The complete digestion process is time‐consuming and critical in terms of stopping the digestion at the right time (stopping it too early can lead to low cell recovery, while stopping it too late can lead to cell damage). Moreover, a long digestion process (>8 hours) might affect the expression of certain genes of interest.^21^, ^22^ In the predigestion and pulverization method, a short digestion (1 hour) is proposed, which will not affect the expression of most genes of interest. In the present study, some variations were also observed in the dCt values of certain genes (Figure 5). These variations might be explained by the differences in the purity of the samples that may have influenced the amplification efficiency of certain primers to a variable extent. Nevertheless a possible effect of the enzyme treatment on the gene expression profile cannot be completely excluded. A previous study, comparing rat NP and AF marker genes between RNA extracted from isolated cells and RNA extracted directly from tissues, did not reveal any significant differences in the relative gene expression levels.^11^ Further experiments would be required to investigate this subject on a whole transcriptome level.

As an alternative method, tissue pulverization in liquid nitrogen followed by RNA extraction in guanidinium thiocyanate‐phenol‐chloroform and further purification of the aqueous phase with silica columns have been reported as a way to eliminate contaminants due to the coprecipitation of ECM components during RNA extraction.^11^ However, in the present study this method led to lower yield and quality compared to predigestion and pulverization.

As for the gene expression, higher Ct values were observed for the endogenous control genes in the pulverization only group, which could be due to low yield and/or the possible presence of contaminants (eg, proteoglycans from ECM, TRIzol carry‐over) which may affect both the reserve transcription and the RT‐PCR. For instance, it has been shown that proteoglycans can act as inhibitors of PCRs.^15^

In conclusion, this new combined method (predigestion and pulverization) improves the yield and quality of the RNA extracted from IVD tissues (NP, inner and outer AF) compared to pulverization alone. As a guideline when a RIN analyzer is not available (and based on the results displayed in Figure 3), the reference values for RNA evaluation with Nanodrop could be set as 20 ng/μL for RNA concentration and 260/280 ratio >1.8. This is achievable even for NP tissue using the predigestion and pulverization method. This method is of importance for gene expression studies on IVD degeneration and regeneration, including the most recent strategies such as rejuvenating the aging disc using stem cells and enhancing the endogenous repair of the disc by injecting an appropriate stimulating factor. Given the similarity in glycosaminoglycan^23^ and collagen^24^ content of IVD tissues across various species, we presume that the proposed RNA isolation method can be applied to IVD samples from different species. Moreover, the results obtained here using young bovine IVDs could be translatable to IVD tissues obtained from older animals. Indeed, with aging the collagen content is increasing at the expense of glycosaminoglycans. Since the latter are the strong contaminants in RNA purification, extraction of RNA from IVD of older animals may not be more challenging compared to younger animals. Nevertheless, direct comparison of all methods among animals of different age groups will be required to address this question. Finally, we also anticipate that this method may be further applied to other tissues rich in proteoglycans.

## Supporting information


**Table S1** Average RNA yield, concentration and purity for the NP, inner AF, and outer AF of bovine discs, according to the method used to isolate the RNA from each sample. NP, nucleus pulposus; iAF, inner annulus fibrosus; oAF, outer annulus fibrosus. Data are represented as mean ± SD; *n* = 3‐6.
**Table S2** RNA concentration and RNA integrity number by Bioanalyzer quantification RNA concentration and 260/280 ratio by Nanodrop for NP, inner AF, and outer AF of bovine discs, according to the method used to isolate the RNA from each sample. Results of both duplicates are provided (n.a., not applicable).
**Table S3** Average Ct values for RT‐PCR of endogenous control genes for the NP, inner AF, and outer AF tissues of bovine discs, according to the method used to isolate the RNA from each sample. Data are represented as mean ± SD; *n* = 3‐6.
**Table S4** Average dCt values for RT‐PCR of genes of interest for the NP, inner AF, and outer AF tissues of bovine discs, according to the method used to isolate the RNA from each sample. Data are represented as mean ± SD; *n* = 3‐6; n.d., gene not detected.
