# Supplementary material for: Isolation of high‐quality RNA from intervertebral disc tissue via pronase predigestion and tissue pulverization
Source: JOR Spine. 2018 Jun 19;1(2):e1017. doi: 10.1002/jsp2.1017 (PMC6686795; doi:10.1002/jsp2.1017)
Supplement: Supplementary file 1 — Table S1 Average RNA yield, concentration and purity for the NP, inner AF, and outer AF of bovine discs, according to the method used to isolate the RNA from each sample. NP, nucleus pulposus; iAF, inner annulus fibrosus; oAF, outer annulus fibrosus. Data are represented as mean ± SD; n = 3‐6. Table S2 RNA concentration and RNA integrity number by Bioanalyzer quantification RNA concentration and 260/280 ratio by Nanodrop for NP, inner AF, and outer AF of bovine discs, according to the method used to isolate the RNA from each sample. Results of both duplicates are provided (n.a., not applicable). Table S3 Average Ct values for RT‐PCR of endogenous control genes for the NP, inner AF, and outer AF tissues of bovine discs, according to the method used to isolate the RNA from each sample. Data are represented as mean ± SD; n = 3‐6. Table S4 Average dCt values for RT‐PCR of genes of interest for the NP, inner AF, and outer AF tissues of bovine discs, according to the method used to isolate the RNA from each sample. Data are represented as mean ± SD; n = 3‐6; n.d., gene not detected. [file JSP2-1-e1017-s001.docx]

**Supporting Information**

**Table 1**: Average RNA yield, concentration and purity for the NP, Inner AF, and Outer AF of bovine discs, according to the method used to isolate the RNA from each sample. NP: nucleus pulposus, iAF: inner annulus fibrosus, oAF: outer annulus fibrosus. Data are represented as mean ± standard deviation; n=3-6.

|  |  | **ng RNA/**  **mg tissue** | **ng RNA/µL** | **260/280** | **260/230** |
| --- | --- | --- | --- | --- | --- |
| **NP** | Complete matrix digestion | 7.29 ± 4.28 | 34.76 ± 20.26 | 1.95 ± 0.13 | 0.42 ± 0.06 |
|  | Pre-digestion and pulverization | 8.82 ± 2.05 | 42.67 ± 6.39 | 1.91 ± 0.15 | 1.84 ± 0.30 |
|  | Pulverization only | 2.53 ± 1.46 | 12.82 ± 7.25 | 1.49 ± 0.12 | 0.68 ± 0.29 |
|  | Pulverization and column | 2.03 ± 1.05 | 9.87 ± 4.30 | 1.61 ± 0.14 | 0.44 ± 0.34 |
| **iAF** | Complete matrix digestion | 2.00 ± 0.29 | 10.35 ± 1.94 | 2.04 ± 0.07 | 0.33 ± 0.05 |
|  | Pre-digestion and pulverization | 15.63 ± 11.76 | 73.37 ± 53.02 | 1.86 ± 0.27 | 1.48 ± 0.37 |
|  | Pulverization only | 3.59 ± 1.57 | 17.16 ± 6.17 | 1.61 ± 0.18 | 1.00 ± 0.41 |
|  | Pulverization and column | 4.63 ± 3.76 | 18.90 ± 14.04 | 1.77 ± 0.13 | 0.68 ± 0.21 |
| **oAF** | Complete matrix digestion | 2.35 ± 0.93 | 11.77 ± 3.28 | 1.94 ± 0.07 | 0.33 ± 0.11 |
|  | Pre-digestion and pulverization | 50.00 ± 26.03 | 253.55 ± 146.33 | 2.11 ± 0.03 | 1.93 ± 0.13 |
|  | Pulverization only | 21.27 ± 14.74 | 119.49 ± 77.23 | 1.91 ± 0.09 | 1.27 ± 0.61 |
|  | Pulverization and column | 4.71 ± 3.67 | 23.78 ± 14.06 | 1.84 ± 0.15 | 1.10 ± 0.27 |

**Table 2**: RNA concentration and RNA Integrity Number by Bioanalyzer quantification RNA concentration and 260/280 ratio by Nanodrop for NP, Inner AF, and Outer AF of bovine discs, according to the method used to isolate the RNA from each sample. Results of both duplicates are provided (n.a. = not applicable).

|  |  | **Bioanalyzer** |  | **Nanodrop** |  |
| --- | --- | --- | --- | --- | --- |
|  |  | **ng RNA/µL** | **RIN** | **ng RNA/µL** | **260/280** |
| **NP** | Complete matrix digestion | 9.8, 2.3 | 8.3, 6.2 | 31.5, 10.1 | 1.75, 1.69 |
|  | Pre-digestion and pulverization | 23.1, 30.8 | 7.2, 7.1 | 37.9, 47.2 | 1.93, 2.09 |
|  | Pulverization only | 1.4, 8.6 | n.a., n.a. | 55.8, 201.7 | 0.92, 1.47 |
|  | Pulverization and column | 5.7, 0.9 | 3.4, 1.5 | 28.1, 14.8 | 1.52, 1.46 |
| **iAF** | Complete matrix digestion | 5.2, 6.3 | 8.7, 6.4 | 21.4, 10.1 | 1.71, 2.04 |
|  | Pre-digestion and pulverization | 31.1, 29.4 | 7.4, 7 | 41.4, 41.9 | 2.09, 2.01 |
|  | Pulverization only | 2.2, 6.8 | n.a., n.a. | 42.1, 96.8 | 0.8, 0.98 |
|  | Pulverization and column | 12.5, 21.6 | 6.2, 2.5 | 20.6, 28.7 | 1.68, 1.80 |
| **oAF** | Complete matrix digestion | 9.0, 6.7 | 7.6, 8.2 | 15.3, 18.1 | 1.92, 1.74 |
|  | Pre-digestion and pulverization | 40.0, 35.2 | 7.4, 8.0 | 72.9, 64.6 | 2.08, 2.06 |
|  | Pulverization only | 34.4, 36.1 | 2.5, 2.2 | 266.3, 249.9 | 1.65, 1.76 |
|  | Pulverization and column | 38.2, 40.7 | 5.9, 2.6 | 80.5, 106.1 | 1.84, 2.02 |

**Table 3**: Average Ct values for RT-PCR of endogenous control genes for the NP, inner AF, and outer AF tissues of bovine discs, according to the method used to isolate the RNA from each sample. Data are represented as mean ± standard deviation; n=3-6.

|  |  | **bGAPDH** | **18S** |
| --- | --- | --- | --- |
| **NP** | Complete matrix digestion | 24.13 ± 0.90 | 16.67 ± 0.54 |
|  | Pre-digestion and pulverization | 24.46 ± 1.20 | 16.55 ± 0.85 |
|  | Pulverization only | 32.76 ± 1.82 | 26.77 ± 2.26 |
|  | Pulverization and column | 28.87 ± 0.64 | 21.38 ± 0.83 |
| **iAF** | Complete matrix digestion | 25.28 ± 0.35 | 17.15 ± 0.37 |
|  | Pre-digestion and pulverization | 25.16 ± 0.58 | 17.53 ± 0.66 |
|  | Pulverization only | 27.48 ± 1.21 | 20.27 ± 2.05 |
|  | Pulverization and column | 27.65 ± 2.10 | 19.50 ± 1.56 |
| **oAF** | Complete matrix digestion | 26.18 ± 0.50 | 17.79 ± 0.20 |
|  | Pre-digestion and pulverization | 24.16 ± 0.57 | 16.65 ± 0.53 |
|  | Pulverization only | 26.63 ± 0.66 | 19.76 ± 0.88 |
|  | Pulverization and column | 26.47 ± 0.67 | 19.27 ± 0.83 |

**Table 4**: Average dCt values for RT-PCR of genes of interest for the NP, inner AF, and outer AF tissues of bovine discs, according to the method used to isolate the RNA from each sample. Data are represented as mean ± standard deviation; n=3-6; n.d. = gene not detected.

|  |  | **COL1** | **COL2** | **ACAN** | **MMP3** | **ADAMTS4** |
| --- | --- | --- | --- | --- | --- | --- |
| **NP** | Complete matrix digestion | 18.49 ± 2.24 | 3.61 ± 0.86 | 9.90 ± 1.01 | 17.40 ± 0.43 | 20.57 ± 0.70 |
|  | Pre-digestion and pulverization | 18.74 ± 1.69 | 3.02 ± 0.49 | 8.97 ± 0.75 | 18.84 ± 1.37 | 20.73 ± 0.85 |
|  | Pulverization only | 12.43 ± 1.47 | 0.06 ± 0.75 | 6.76 ± 2.08 | n.d. | n.d. |
|  | Pulverization and column | 16.92 ± 1.79 | 2.63 ± 0.72 | 8.93 ± 1.12 | 16.77 ± 1.19 | 16.91 ± 1.32 |
| **iAF** | Complete matrix digestion | 13.63 ± 2.21 | 3.07 ± 0.72 | 9.16 ± 0.11 | 16.29 ± 0.51 | 19.52 ± 1.97 |
|  | Pre-digestion and pulverization | 13.09 ± 3.56 | 3.77 ± 0.64 | 10.26 ± 0.54 | 16.70 ± 0.85 | 17.32 ± 2.13 |
|  | Pulverization only | 11.09 ± 2.28 | 2.97 ± 1.26 | 9.70 ± 1.36 | 16.45 ± 2.50 | 17.37 ± 1.27 |
|  | Pulverization and column | 11.64 ± 1.41 | 3.71 ± 0.62 | 10.29 ± 0.85 | 16.90 ± 1.33 | 17.79 ± 1.57 |
| **oAF** | Complete matrix digestion | 8.25 ± 0.21 | 6.88 ± 2.54 | 12.34 ± 1.24 | 14.12 ± 1.91 | 13.79 ± 1.19 |
|  | Pre-digestion and pulverization | 8.81 ± 0.20 | 9.06 ± 2.40 | 13.43 ± 1.16 | 12.94 ± 1.11 | 13.92 ± 0.50 |
|  | Pulverization only | 8.56 ± 0.49 | 7.92 ± 2.08 | 12.97 ± 1.12 | 10.56 ± 2.25 | 13.73 ± 0.88 |
|  | Pulverization and column | 8.52 ± 0.26 | 8.15 ± 2.06 | 13.74 ± 1.50 | 12.58 ± 1.44 | 13.99 ± 0.87 |
